# Efficacy and safety of an alpha 7-nicotinic acetylcholine receptor agonist, VQW-765, in subjects with performance anxiety: randomised, double-blind, placebo-controlled trial

**DOI:** 10.1192/bjp.2025.84

**Published:** 2025-07

**Authors:** Yunsheng He, Christos M. Polymeropoulos, Michael A. Mohrman, Sunny O. Truslow, Changfu Xiao, Yukun Wu, Gunther Birznieks, Mihael H. Polymeropoulos

**Affiliations:** Vanda Pharmaceuticals, Washington, DC, USA

**Keywords:** Performance anxiety, social anxiety disorder, alpha-7 nicotinic acetylcholine receptor, Trier Social Stress Test

## Abstract

**Background:**

Despite the high prevalence of social and performance anxiety, current treatments do not meet the full needs of patients. Development of novel anxiolytics with rapid onset of action for on-demand treatment of social and performance anxiety is an active area of clinical research.

**Aims:**

To examine the anxiolytic effect of VQW-765, an α7-nAChR agonist, in subjects with performance anxiety.

**Method:**

We conducted a randomised, double-blind, placebo-controlled trial of 230 adults with a history of public speaking anxiety. Participants were randomly assigned to receive a single oral dose of 10 mg VQW-765 (*n* = 116) or placebo (*n* = 114), followed by a Trier Social Stress Test (TSST). Anxiety levels were assessed by the Subjective Units of Distress Scale (SUDS). Heart rate was monitored during the TSST. Plasma concentration of VQW-765 was measured after the TSST.

**Results:**

Subjects receiving VQW-765 showed a trend of improvement in intensity of anxiety, as measured by the SUDS, during the performance phase of a TSST compared with placebo (*P* = 0.1443). Females showed a larger magnitude and significant response to VQW-765 (*P* = 0.034). The pharmacokinetic/pharmacodynamic analysis observed an inverted U-shaped exposure–response relationship. Subjects in the middle 50% quantiles of VQW-765 plasma concentration showed significant improvement in the SUDS rating compared with placebo (*P* = 0.033); however, subgroup analysis revealed this was true only for females (*P* = 0.005). VQW-765 was safe and well tolerated.

**Conclusions:**

This is the first study showing anxiolytic effect of an α7-nAChR agonist in humans. VQW-765 is a promising candidate to be developed for on-demand treatment of social anxiety disorder.

Social anxiety disorder (SAD) is a common and debilitating psychiatric condition with a lifetime prevalence of about 13%.^1^ Females are more likely than males to develop SAD and have more severe clinical symptoms and greater subjective distress than males.^2^ SAD is characterised by marked and persistent fear of negative social evaluation and avoidance of such social situations. People with SAD often have impairments in occupational, educational, social or other important areas of functioning, and have a high rate of comorbidity with other psychiatric disorders including major depressive disorder.^1,2^

Performance anxiety is one of the major symptoms of SAD, which is defined as an excessive fear of speaking or performing in public situations.^1^ It is estimated that over 80% of SAD patients experience performance anxiety and approximately 40% of them experience severe performance anxiety.^3^ Public speaking anxiety is the most reported type of performance anxiety, with an estimated total prevalence rate of 20% of the general population.^3,4^ Because performance anxiety occurs only in specific situations, it would be helpful to have a treatment option that can be used on an as-needed basis to manage the acute symptoms. However, no such treatment has been approved by regulatory authorities to date.

Beta-adrenergic receptor blockers and benzodiazepines have been used off-label to combat the symptoms of performance anxiety, but their efficacy and safety have never been confirmed in well-controlled clinical trials.^5,6^ Selective serotonin reuptake inhibitors have been approved for the treatment of anxiety disorders, including SAD,^7,8^ but cannot be used for on-demand treatment due to their slow onset of action. Development of novel anxiolytics with rapid onset of action for on-demand treatment of SAD is an active area of clinical research. PH94B, a neuroactive steroid nasal spray, significantly reduced performance anxiety in women with SAD in a phase 2 study,^9^ and cannabidiol showed a significant effect on reducing performance anxiety in three independent clinical studies.^10–12^

The Trier Social Stress Test (TSST) is regarded as the gold standard in simulating a stressful social situation and is commonly used in studies of physiological and psychological responses to social-evaluative threats, as well as in determining the effectiveness of an acutely acting agent for social and performance anxiety.^9–13^ A sex difference in response to the TSST was reported, with females experiencing more subjective stress and greater increase in heart rate than males during a TSST.^14^ In the present study, the TSST involving a public speaking challenge was used to examine the treatment effect of VQW-765 in subjects with performance anxiety.

## Targeting the α7-nAChR pathway for treating anxiety

VQW-765, formerly known as AQW051, is a selective agonist for the alpha-7 nicotinic acetylcholine receptor (α7-nAChR) and has demonstrated a strong effect on cognitive function in animal models.^15^ α7-nAChR is a homopentameric nAChR with both ionotropic and metabotropic functioning, and is highly expressed in the brain and immune cells, including microglia.^16^ The α7-nAChR signalling pathway plays important roles in neurotransmitter release, cognitive functioning and the cholinergic anti-inflammatory response. Genetic abnormalities in the *CHRNA7* gene that encodes α7-nAChR have been implicated in several neurological and psychiatric disorders.^16^ α7-nAChR was also reported to be involved in the acute stress response by modulating the hypothalamic–pituitary–adrenal axis.^17^

In addition to its effect on cognition, VQW-765 has shown a strong anxiolytic effect in preclinical studies. A single oral dose of VQW-765 significantly increased the duration of social contacts in rats.^15^ The effect of VQW-765 is comparable to the known anxiolytic agent chlordiazepoxide; the dose–response relationship was inverted U-shaped, probably due to prolonged receptor desensitisation at higher doses. VQW-765 has a rapid onset of action and represents a promising candidate for development as a medication that can be used on an as-needed basis to manage acute symptoms for subjects with social and performance anxiety. To replicate the preclinical findings, we conducted a proof-of-concept study to examine the anxiolytic effect of VQW-765 in subjects with performance anxiety.

## Method

### Clinical trial design

The efficacy and safety of VQW-765 were assessed in a double-blind, placebo-controlled, randomised trial (no. NCT04800237) of 230 adults with a history of performance anxiety at 15 sites in the USA, from February 2021 until August 2022. The authors assert that all procedures contributing to this work comply with the ethical standards of the relevant national and institutional committees on human experimentation, and with the Helsinki Declaration of 1975 as revised in 2013. All procedures involving human subjects were approved by the central independent review board (IRB) Advarra 144 (no. Pro00047343), and by local IRBs NYU (deferred to Advarra central IRB – no study ID) and UCF 145 (no. STUDY00003123).

Participants were randomly assigned to groups by a centralised, web-based, validated system and given a single oral dose of 10 mg VQW-765 (*n* = 116) or matching placebo (*n* = 114), followed by a TSST. Anxiety levels were assessed by the Subjective Units of Distress Scale (SUDS).^18^ The primary objectives were to assess the effect of a single oral dose of 10 mg VQW-765 relative to placebo on the SUDS rating during the performance phase of a TSST in the intention-to-treat (ITT) and female populations, the Clinician Global Impression of Change (CGI-C) scale and the Patient Global Impression of Change (PGI-C) scale in the ITT population following the TSST, and the exposure–response relationship. The secondary objectives were to assess the effect of VQW-765 relative to placebo on the SUDS rating during the performance of a TSST in subjects with baseline Liebowitz Social Anxiety Scale (LSAS) score ≥60, and the safety and tolerability of VQW-765 during the study.

Date first patient screened: 23 February 2021

Date first patient enrolled: 29 March 2021

Date last patient completed testing: 2 August 2022.

### Participants

Eligible participants should be 18–70 years of age, non-smokers and non-nicotine users, have elevated public speaking anxiety as measured by Public Speaking Anxiety Scale (PSAS) total score ≥60 and have a 17-item Hamilton Depression Rating Scale (HAM-D) score of ≤18. Subjects should be excluded if they have a history of bipolar disorder, schizophrenia, psychosis, seizures, delusional disorders, obsessive–compulsive disorder, post-traumatic stress disorder, eating disorder or substance or alcohol use disorder in their medical records. Subjects should also be excluded if they had any concurrent psychotherapy in the past 6 months, or ongoing psychotherapy or any concurrent psychotropic medication in the past 2 months.

### Procedures

At visit 2, participants were asked for a baseline SUDS rating, then randomised and given a capsule of 10 mg VQW-765 or placebo. Two hours later, participants were instructed to prepare for a 5 min speech for a mock job interview (resting phase). Following 3 min of preparation (anticipation phase), participants delivered the speech in front of an interview panel with two clinical staff (performance phase). The SUDS rating was collected each 60 s during the TSST.

### Assessments

SUDS is a self-reported rating scale from 0 to 100, and was used to measure the intensity of anxiety during the TSST.^18^ CGI-C and PGI-C were administered following the TSST. LSAS is a 24-item, self-reported rating scale used to assess the severity of social anxiety symptoms, and was administered at screening. Heart rate was continuously measured using the ePatch device (Philips-BioTelemetry) during the TSST. Heart rate variability (HRV) was calculated by the root mean square of successive differences between heartbeats (RMSSD) method.^19^

### Plasma drug concentration assessment

A single blood sample was collected from all participants following the TSST. The plasma concentration of VQW-765 was determined by a liquid chromatography–tandem mass spectrometry method developed and validated by QPS (Newark, DE, USA).

### Statistical analysis

The average anxiety rating as measured by SUDS during the performance of a TSST was analysed using a mixed-effect model repeated measurement (MMRM). The MMRM model included the fixed, categorical effects of treatment group, phase, treatment group-by-phase interaction and pooled site. This analysis was performed in both the ITT and female populations. CGI-C and PGI-C were analysed using an analysis of variance (ANOVA) model, which included the fixed treatment group and pooled site. Exposure–response to VQW-765 was assessed by the SUDS rating during the performance of a TSST by quantiles of VQW-765 plasma concentration in the ANOVA model. The multiplicity of multiple endpoints was adjusted by the Hommel method. The planned sample size of 110 subjects per arm provides 85% power to detect a mean difference of 12 points in the mean SUDS score, assuming a standard deviation of 28 in each group. All data processing, summarisation and analyses were performed using SAS version 9.3 or higher (SAS Institute Inc., Cary, NC, USA).

## Results

### Participants

Of the 459 subjects screened, 230 were enrolled and randomised to either VQW-765 (*n* = 116) or placebo (*n* = 114) treatment groups. All randomised subjects completed the study and were included in the analysis (Supplementary Fig. 1). Randomised subjects had a mean age of 41.4 years and 69.1% of them were female. At baseline, the mean measures of the 17-item HAM-D, PSAS and LSAS were 4.3, 70.2 and 83.3, respectively (Table 1). There were no significant differences between treatment groups in regard to demographic characteristics and baseline measures.

**Table 1:** Demographics and key baseline measures

All randomised subjects	VQW-765	Placebo	Total
	(*n* = 116)	(*n* = 114)	(*n* = 230)
Sex, *n* (%)			
Female	82 (70.69)	77 (67.54)	159 (69.13)
Male	34 (29.31)	37 (32.46)	71 (30.87)
Age (years)			
Mean (s.d.)	41.04 (15.23)	41.72 (15.20)	41.38 (15.19)
Race, *n* (%)			
White	66 (56.90)	59 (51.75)	125 (54.35)
Black or African-American	30 (25.86)	31 (27.19)	61 (26.52)
Asian	13 (11.21)	19 (16.67)	32 (13.91)
American-Indian or Alaska Native	3 (2.59)	1 (0.88)	4 (1.74)
Native Hawaiian or other Pacific Islander	1 (0.86)	1 (0.88)	2 (0.87)
Others	3 (2.59)	3 (2.63)	6 (2.61)
Body mass index (kg/m^2^)			
Mean (s.d.)	28.10 (6.24)	29.10 (7.32)	28.60 (6.80)
17-item HAM-D			
Mean (s.d.)	4.18 (4.06)	4.32 (4.28)	4.25 (4.16)
PSAS			
Mean (s.d.)	69.99 (6.44)	70.39 (6.79)	70.19 (6.60)
LSAS			
Mean (s.d.)	83.14 (24.65)	83.50 (26.31)	83.32 (25.43)
LSAS ≥60, *n* (%)	97 (83.62)	92 (80.70)	189 (82.17)

HAM-D, Hamilton Depression Rating Scale; PSAS, Public Speaking Anxiety Scale; LSAS, Liebowitz Social Anxiety Scale.

A formal diagnosis of SAD was not required for the present study. Of the 230 subjects randomised, only 4 (3 ongoing and 1 prior) had a documented history of SAD. Eligible participants were required to be suffering from elevated public speaking anxiety, as determined by PSAS total score ≥60. Most subjects enrolled in the study (82.2%) exhibited moderate to severe social anxiety symptoms, as defined by LSAS total score ≥60 at baseline (Table 1).

### Primary efficacy on SUDS

**Fig. 1:**
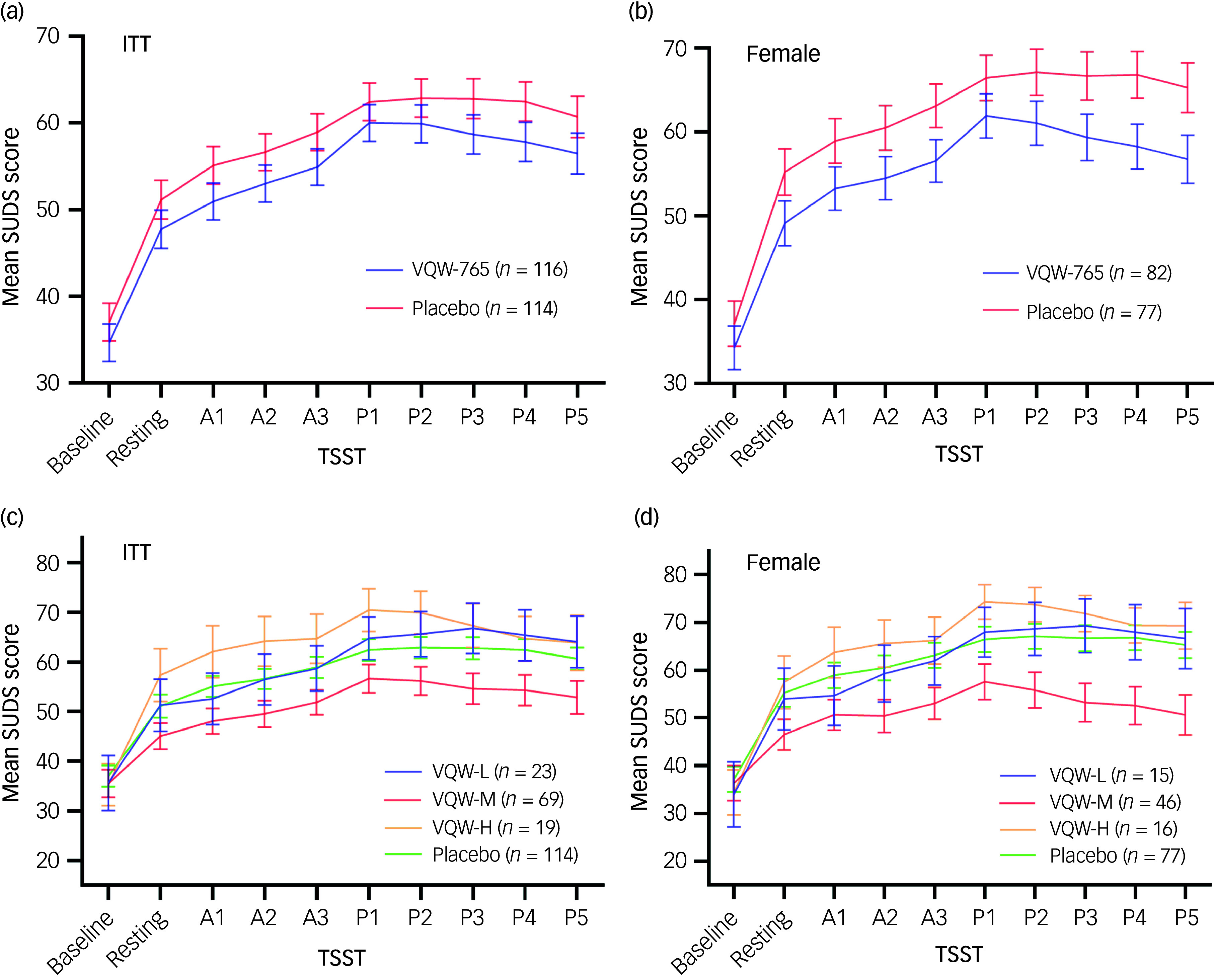
Mean SUDS score for patients receiving VQW-765 or placebo during the TSST. (a) and (b) Curves representing mean SUDS score (±s.e.m.) at each time point of the TSST for groups treated with VQW-765 or placebo in the (a) ITT and (b) female populations. VQW-765 concentration in plasma was measured following the TSST. VQW-L, VQW-M and VQW-H indicate low, moderate and high exposure, respectively. (c) and (d) Mean SUDS scores (±s.e.m.) at each time point of the TSST for the groups with varying exposure of VQW-765 and placebo in the (c) ITT and (d) female populations are illustrated. Baseline, pre-dose; resting, resting phase (task introduction); A1–3, anticipation phase; P1–5, performance phase; SUDS, Subjective Units of Distress Scale; TSST, Trier Social Stress Test; ITT, intention-to-treat.

The principal assessment for the effectiveness of VQW-765 was the self-reported SUDS rating in the ITT and female populations. Subjects receiving VQW-765 showed a trend of improvement in intensity of anxiety, as measured by SUDS, during the performance phase of a TSST, compared with placebo (*P* = 0.1443) (Fig. 1(a) and Table 2). Females (69.1% of total participants) receiving VQW-765 showed a higher magnitude and significant response to VQW-765 (*P* = 0.034) compared with placebo (Fig. 1(b) and Table 2). In contrast, males receiving VQW-765 did not show any improvement on the SUDS rating compared with placebo (Supplementary Fig. 2).

**Table 2:** Efficacy analyses

Population	Assessment	TSST	VQW-765 (*n* = 116)		Placebo (*n* = 114)		Difference (95% CI)	*P*-value
			Mean	s.d.	Mean	s.d.		
ITT	SUDS	Pre-dose	34.66	23.20	37.02	23.42		
		Resting	47.76	23.07	51.15	24.78	−2.8 (−8.70, 3.14)	0.1775
		Anticipation	52.96	22.25	56.90	21.90	−3.3 (−8.83, 2.23)	0.1206
		Performance^a^	58.6	23.96	62.26	22.40	−3.2 (−9.13, 2.73)	0.1443
	CGI-C	Post-TSST^a^	3.6	1.12	3.6	1.06	−0 (−0.29, 0.23)	0.4038
	PGI-C	Post-TSST^a^	3.4	1.14	3.5	1.08	−0 (−0.33, 0.25)	0.3993

			VQW-765 (*n* = 82)		Placebo (*n* = 77)			*P*-value
Female	SUDS	Pre-dose	34.27	23.88	37.14	23.72		
		Resting	49.15	22.94	55.26	25.59	−5.9 (−13.51, 1.74)	0.0645
		Anticipation	54.80	22.33	60.88	22.33	−5.8 (−12.79, 1.24)	0.0531
		Performance^a^	59.55	24.63	66.42	22.41	−6.9 (−14.26, 0.52)	0.0342

			VQW-765 (*n* = 56)		Placebo (*n* = 114)			*P*-value
ITT, >50% quantile	SUDS	Pre-dose	35.89	23.95	37.02	23.42		
		Resting	46.25	22.04	51.15	24.78	−4.9 (−12.61, 2.81)	0.1059
		Anticipation	50.89	20.82	56.9	21.90	−6.0 (−13.06, 1.04)	0.0472
		Performance^a^	55.36	24.52	62.26	22.40	−6.9 (−14.28, 0.47)	0.0332

			VQW-765 (*n* = 38)		Placebo (*n* = 77)			*P*-value
Female, 50% quantile	SUDS	Pre-dose	36.58	25.39	37.14	23.72		
		Resting	47.63	21.74	55.26	25.59	−7.6 (−17.16, 1.89)	0.0577
		Anticipation	53.07	21.30	60.88	22.33	−7.8 (−16.46, 0.85)	0.0384
		Performance^a^	54.37	25.19	66.42	22.41	−12.0 (−21.07, −3.03)	0.0046

			VQW-765 (*n* = 97)		Placebo (*n* = 92)			*P*-value
ITT, LSAS ≥60	SUDS	Pre-dose	37.01	23.01	39.78	23.95		
		Resting	49.28	23.68	52.97	24.70	−1.8 (−8.51, 4.96)	0.3018
		Anticipation	54.05	23.04	57.62	22.24	−1.6 (−7.92, 4.69)	0.3070
		Performance^b^	59.67	24.72	62.85	22.80	−1.5 (−8.21, 5.26)	0.3329

SUDS, Subjective Units of Distress Scale; TSST, Trier Social Stress Test; CGI-C, Clinician Global Impression of Change; PGI-C, Patient Global Impression of Change; ITT, intention-to-treat; 50% quantile, middle 50% quantiles of VQW-765 plasma concentration; LSAS, Liebowitz Social Anxiety Scale. Difference (95% CI), least-square mean difference (95% CI).

a. Primary efficacy analysis.

b. Secondary efficacy analysis.

In line with previous studies, females reported much higher anxiety than males during the TSST when treated with placebo (Supplementary Fig. 3(a)). The results suggest that lack of efficacy in males could be due to the sex difference in response to the TSST.

### Exposure–response relationship

To gain more insight into the dynamic action of VQW-765 during the psychosocial stress test, VQW-765 plasma concentration was measured, with a broad range of exposure being observed (Supplementary Fig. 4). To assess the inverted U-shaped dose–response relationship observed in preclinical studies, we performed an efficacy analysis by quantiles of VQW-765 plasma concentration. Subjects in the middle 50% showed significant improvement in SUDS rating compared with placebo (*P* = 0.033), whereas those treated with VQW-765 in the first and fourth quartiles showed a performance similar to placebo (*P* = 0.405 and *P* = 0.460, respectively). Females in the middle 50% showed a stronger response compared with placebo (*P* = 0.005) (Table 2). VQW-765 plasma concentrations in the middle 50% ranged from 1.57 to 6.32 pmol/mL. Based on the corresponding SUDS rating, the range of efficacious plasma concentrations can be refined to 1.5–8.0 pmol/mL (Supplementary Fig. 4). A post hoc analysis showed that performance anxiety was significantly alleviated in subjects with efficacious exposure of VQW-765 compared with placebo (*P* = 0.020), but only for females (*P* = 0.003) (Fig. 1(c) and (d) and Supplementary Table 1). Males with efficacious exposure of VQW-765 did not show any improvement compared with placebo (Supplementary Fig. 5).

### Other primary efficacy measures

PGI-C and CGI-C were included in the primary efficacy analysis, because there is no consensus on the preferred means of evaluating treatment response following a TSST. No significant differences between VQW-765 and placebo were observed in either PGI-C (*P*= 0.399) or CGI-C (*P* = 0.404) (Table 2). The post hoc analysis for subjects with efficacious exposure of VQW-765 also did not reveal any significant difference between VQW-765 and placebo in either PGI-C (*P* = 0.5525) or CGI-C (*P* = 0.4343) (Supplementary Table 1). These results suggest that PGI-C and CGI-C are inadequate assessments in the evaluation of treatment effect in a clinical study with a single TSST procedure.

### Secondary efficacy measures

Approximately 82% of participants had moderate to severe social anxiety symptoms at baseline, for whom we performed an efficacy analysis. The response to VQW-765 in this subset was similar to that in the overall study population (Table 2 and Supplementary Table 1). Within this group, performance anxiety was significantly reduced in those with efficacious exposure of VQW-765 (*P* = 0.015) as compared with placebo (Supplementary Table 1). However, subgroup analysis revealed that this was the case only for females (*P* = 0.005), with no significant effect found for males.

### HRV

To better understand the mechanism of action of VQW-765, HRV change in response to the TSST was explored. Of the 230 subjects enrolled, 141 had synchronised heart rate and SUDS data and were included in the analysis; of these 141 subjects, 73% were female. HRV was calculated by RMSSD, which is the most frequently used time domain method and is especially useful in the study of short-term variation in heart rate.

**Fig. 2:**
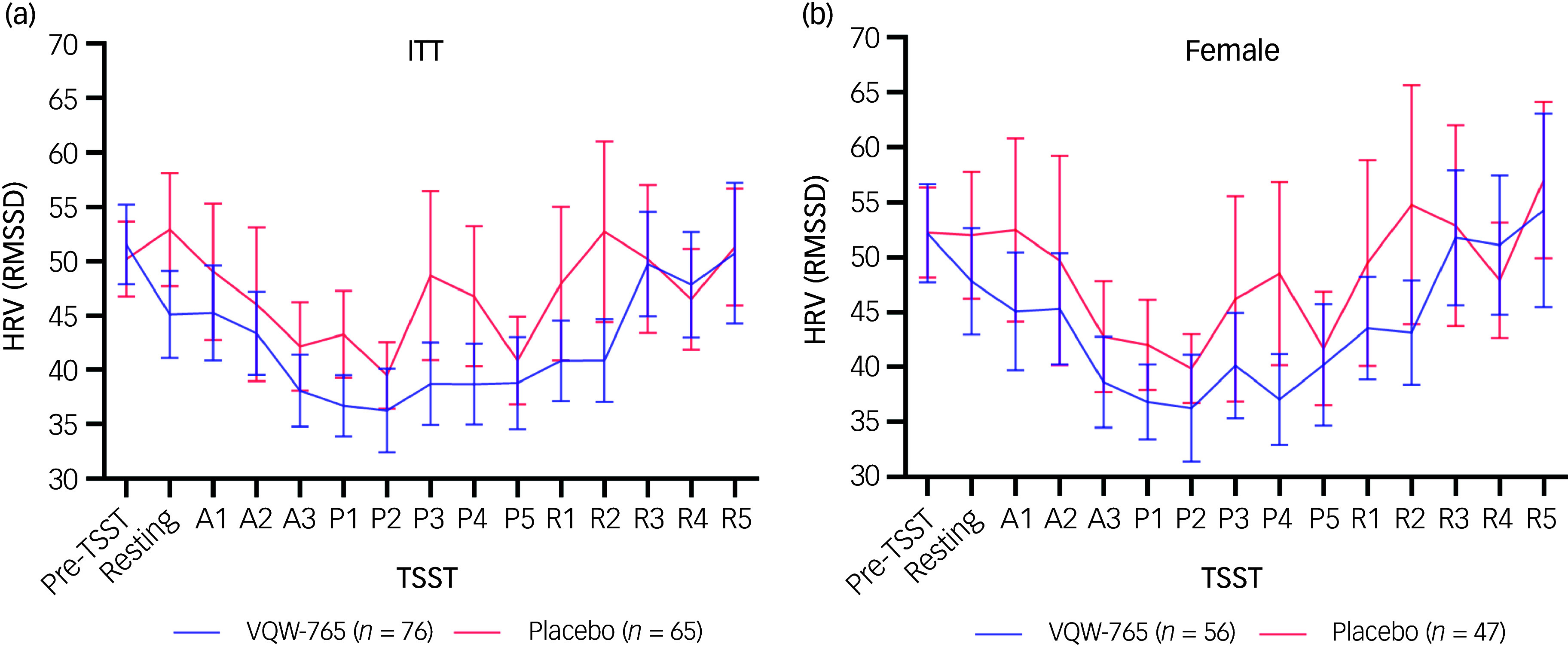
HRV changes during the TSST. (a) and (b) HRV changes during the TSST for patients with synchronised SUDS and heart rate data in the (a) ITT and (b) female populations are illustrated. Pre-TSST, post dose but still in waiting room; resting, resting phase (task introduction); A1–3, anticipation phase; P1–5, performance phase; R1–5, recovery phase following public speaking challenge; RMSSD, root mean square of successive differences between heartbeats; HRV, heart rate variability; TSST, Trier Social Stress Test; SUDS, Subjective Units of Distress Scale; ITT, intention-to-treat.

The response to treatment as measured by the SUDS rating was similar between the subset with synchronised heart rate and SUDS data and the overall study population (Supplementary Fig. 6(a) and (b)). Overall, subjects receiving VQW-765 tended to show a greater decline in HRV than those on placebo in response to the acute stressor (Fig. 2(a) and (b)). In addition, subjects receiving VQW-765 showed a rapid decline in HRV, while those on placebo showed a delayed cardiovascular response to the stressor. HRV recovered to baseline levels in both treatment groups 5 min following the TSST. The recovery curve was relatively steady in subjects receiving VQW-765, but fluctuated in those on placebo (Fig. 2(a) and (b)).

### Safety

The safety analysis included all subjects enrolled in the study (*N* = 230). Overall, there were 13 treatment emergent adverse events (TEAEs) in the placebo group and 6 in the VQW-765 group (Supplementary Table 2). The most common TEAE reported in the study was headache (2 [1.7%] VQW-765 *v*. 4 [3.5%] placebo). No serious adverse events were reported during the study. VQW-765 was found to be safe and well tolerated.

## Discussion

Accumulating evidence from preclinical studies suggests that activation of α7-nAChR signalling results in suppression or prevention of anxiety.^20–22^ The present study is the first to show an anxiolytic effect of an α7-nAChR agonist in a placebo-controlled clinical trial. A formal diagnosis for SAD was not required for the study. Only four participants had SAD in their medical history. The anxiolytic effect of VQW-765 was examined in 230 adults with elevated public speaking anxiety, as determined by self-reported PSAS score ≥60. PSAS is a reliable and valid measure used to assess cognitions, behaviours and physiological manifestations of speech anxiety. This scale was employed in previous clinical studies (nos. NCT03885414, NCT03743571 and NCT04396392). Interestingly, most participants in the study (82.2%) exhibited moderate to severe social anxiety symptoms, as defined by LSAS total score ≥60 at baseline. LSAS is the instrument most frequently used to assess the severity of SAD in clinical research; previous studies showed that LSAS could be used as a cost-effective tool to screen SAD in a clinical setting.^23^ LSAS total score ≥60 was reported to be an optimal cut-off value for generalised SAD,^24^ and it is therefore likely that most participants in our study may have had underlying SAD although never previously formally diagnosed. The data suggest that SAD is probably highly underdiagnosed, which is in line with previous research.^25,26^ SAD patients often believe that social anxiety symptoms are part of their personality and therefore cannot be changed.^27^ Less than a fifth of them have ever contacted a professional concerning their social fears, and only about a third of individuals with a lifetime history of SAD reported ever receiving treatment.^25,26^

VQW-765 significantly reduced the intensity of anxiety in females, but not in males, as compared with placebo during the TSST. It is known that there is a sex difference in response to a psychosocial stressor such as TSST.^14^ Typically, females report more anxiety and have a greater increase in heart rate than males during the TSST. Consistent with previous studies, our data showed that females had significantly higher SUDS rating than males during the TSST, including the resting phase (55.3 *v*. 42.7), the anticipation phase (60.9 *v*. 48.7) and the performance phase (66.4 *v*. 53.6), when treated with placebo (Supplementary Fig. 3(a)). Females also had a greater increase in heart rate than males during the TSST (Supplementary Fig. 3(b)). These results suggest that the TSST, as a psychosocial stressor implemented in clinical research, could be a less robust tool for assessment of acute subjective stress in males. Further studies are required to determine the utility of TSST in drug development for males.

The present study was conducted during the COVID-19 pandemic. Although mask wearing was not required in the study protocol, most participants (95%) wore face masks during the TSST procedure, which introduced an unknown variable to the clinical readouts. Mask wearing has been reported to reduce social anxiety symptoms for individuals with SAD when interacting with others or performing in social situations.^28^ To assess the potential impact of mask wearing on stress reactivity during the TSST, we compared our study with a previous study that used a similar TSST procedure and study population. That previous study focused on women with SAD with baseline LSAS score ≥60; average SUDS rating during the performance of a TSST was 80.7 when treated with placebo.^9^ In contrast, SUDS rating was 67.0 in females with baseline LSAS score ≥60 and treated with placebo in our study. These data suggest that mask wearing could significantly reduce stress reactivity during the TSST and potentially reduce the effect of VQW-765 over placebo. Moreover, we performed a post hoc analysis to assess treatment effect in those who did not wear a mask during the TSST. As shown in Supplementary Fig. 7, subjects receiving VQW-765 were significantly less anxious than those receiving placebo, with a large effect size during the TSST. These data support the hypothesis of the effect of mask wearing on treatment response, but the limited sample size in this subset population is a limitation on the analysis.

α7-nAChR features rapid desensitisation during the activation cycle, to prevent excess calcium from entering the cells.^16^ α7-nAChR agonists usually display an inverted U-shaped dose–response curve because of dose-dependent receptor desensitisation. The nature of this curve presents a significant challenge to clinical development for α7-nAChR agonists, because it is not realistic to test a broad range of doses in patients to reduce the risk of prolonged receptor desensitisation. VQW-765 demonstrated a clear pattern of inverted U-shaped dose–response in preclinical studies (data not published). At the maximally effective dose in rats (1 mg/kg), the efficacious plasma concentration of VQW-765 was centred at 1–5 pmol/mL. In the present study, we replicated this curve in humans and identified a window of efficacious plasma concentration of VQW-765 in the treatment of performance anxiety (1.5–8.0 pmol/mL). These results further support the therapeutic hypothesis and provide critical information towards improving future study designs. Interestingly, the window of efficacious plasma concentration of VQW-765 in humans is within a range similar to that observed in rats. The concordance of efficacious plasma concentration between rodents and non-human primates has been reported in previous studies for other α7-nAChR agonists, including BMS-933043 and EVP-6124.^29,31^

The autonomic nervous system (ANS) can be dysregulated in anxiety disorders. Individuals suffering from chronic anxiety tend to have lower HRV compared with healthy subjects during resting-state condition.^32^ However, it remains largely unknown how HRV changes in response to an acute psychosocial stressor, and whether HRV change can be modified by pharmacological intervention. One recent study compared HRV in response to an acute stressor between individuals with chronic anxiety and healthy controls, and reported a different pattern in change of HRV between the two groups.^33^ When the stress test was initiated, HRV rapidly decreased in response to the stressor in healthy controls, while HRV decline was delayed in anxious individuals. The authors hypothesised that the observed difference in the change of HRV was due to a delayed or blunted cardiovascular response to the acute stressor in anxious individuals according to the Generalised Unsafety Theory of Stress (GUTS).^34^ Basically, the stress response is chronically inhibited in healthy subjects if safety is perceived, whereas it is chronically activated in anxious individuals because of difficulties in detecting safety.^34^ In our study, subjects receiving placebo showed a delayed cardiovascular response at the beginning of the TSST, which is similar to that of anxious individuals in the published study. In contrast, subjects receiving VQW-765 showed a rapid decline in HRV in response to the TSST, which is similar to the healthy controls in the published study. A brief and rapid decrease in HRV during a stress test is a sign that the body is responding to the stressor and that the ANS is functioning properly. Once the stressor is resolved, HRV will return to normal. Interestingly, a distinct pattern of HRV recovery between treatment groups was observed. HRV started to increase during the performance phase and returned to baseline levels at the end of the recovery in both treatment groups, but the increase in HRV was steady in subjects receiving VQW-765 while fluctuated in those on placebo. To our knowledge, this is the first study to report differential patterns of HRV in response to an acute stressor in a placebo-controlled clinical trial. Further studies are required to confirm these findings, and to assess whether the differences in HRV change during the TSST may suggest a treatment effect of VQW-765 or serve as a biomarker of treatment response.

The α7-nAChR function in regulation of the brain circuits is complex and could be location, time and context dependent.^35^ In general, activation of α7-nAChR signalling reduces glutamatergic and increases GABAergic transmission through both pre- and postsynaptic mechanisms, supporting a critical role of the receptor in modulating the two opposing classes of neurons that both have behavioural consequences.^36^ Interestingly, BNC210, an α7-nAChR negative-allosteric modulator, also showed anxiolytic effect in patients with generalised anxiety disorder.^37^ It is possible that the observed anxiolytic effect of VQW-765 and BNC210 could be driven by rebalancing of the excitatory and inhibitory signallling pathways; imbalance of these two opposing pathways could lead to a hyper-reactive nervous system and increased risk of anxiety disorders.

There are limitations associated with this study. First, a formal diagnosis for SAD was not required. To fulfil the unmet need in the treatment of SAD, a formal diagnosis for SAD should be implemented in future studies. Second, most participants wore face masks during the TSST procedure, which introduced an unknown variable to the treatment effect of VQW-765.

In conclusion, this is the first time that an α7-nAChR agonist has been shown to exert an anxiolytic effect in a placebo-controlled clinical study. Females with efficacious exposure of VQW-765 demonstrated significant and clinically meaningful improvement in the intensity of anxiety during the TSST. VQW-765 was found to be safe and well tolerated, and no serious adverse events were reported. VQW-765 has potential to transform the treatment strategy for social and performance anxiety and warrants further investigation.

## Supporting information

He et al. supplementary material 1 He et al. supplementary material

He et al. supplementary material 2 He et al. supplementary material

## Data Availability

The data that support the findings of this study are available from the corresponding author (Y.H.) upon reasonable request.

## Supplementary material

The supplementary material can be found online at https://doi.org/10.1192/bjp.2025.84

## Previous presentation

The study results have been presented in the Pharmaceutical Pipeline Session of the 2023 American Society of Clinical Psychopharmacoloyg (ASCP) Annual Meeting (https://pmg.joynadmin.org/documents/1005/645b9b91b2007421627e7bc2.pdf).
