# Supplementary material for: Efficacy and safety of an alpha 7-nicotinic acetylcholine receptor agonist, VQW-765, in subjects with performance anxiety: randomised, double-blind, placebo-controlled trial
Source: Br J Psychiatry. 2025 Jul;227(1):473–80. doi: 10.1192/bjp.2025.84 (PMC12278049; doi:10.1192/bjp.2025.84)
Supplement: He et al. supplementary material 2 — He et al. supplementary material [file S0007125025000844sup002.docx]

**Efficacy and Safety of an Alpha 7 Nicotinic Acetylcholine Receptor Agonist, VQW-765, in Subjects with Performance Anxiety: Randomized, Double-Blind, Placebo-Controlled Trial**

**Supplemental Figures.**

**Figure S1: Flowchart of the study cohort.**

All randomized subjects completed the study and were included in the analysis. The intention-to-treat (ITT) population is the same as the overall study population.

**Figure S2: Mean SUDS score for male patients receiving VQW-765 or placebo during the TSST.**


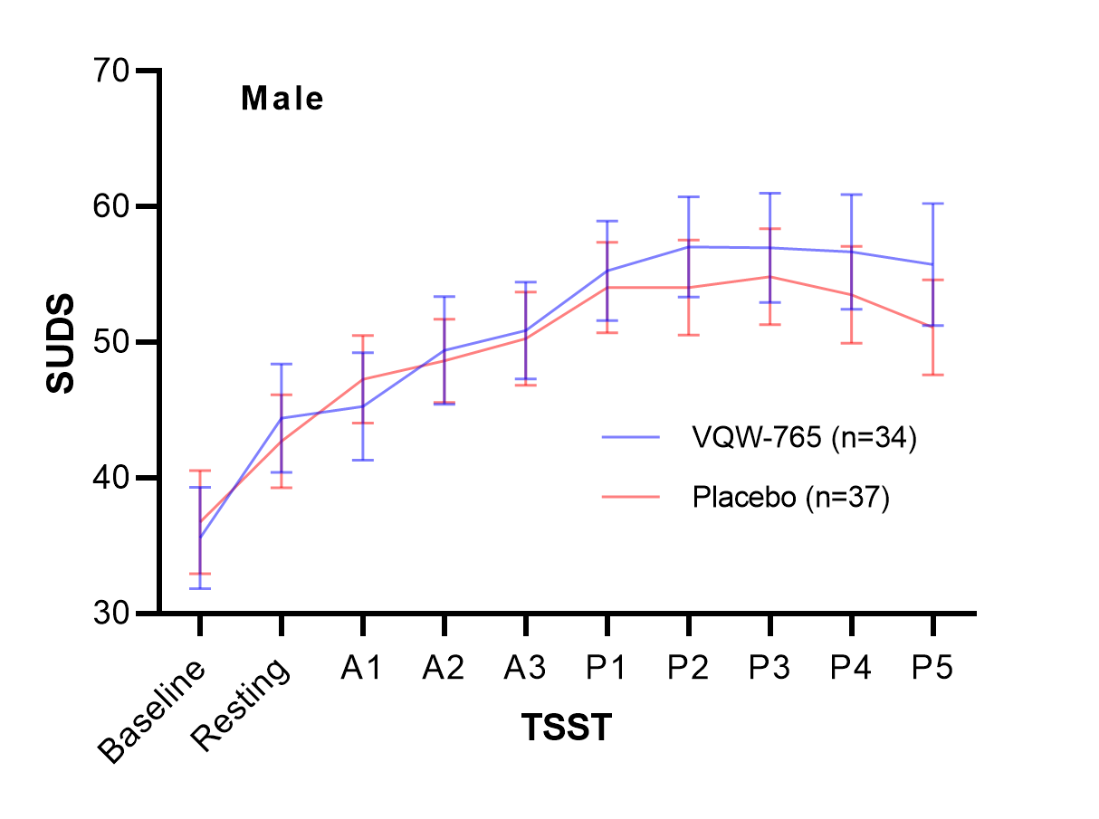


Curves represent mean SUDS score (± SEM) in each timepoint of the TSST for groups treated with VQW-765 or placebo in male patients. Baseline = pre-dose phase; Resting = resting phase (task introduction); A1-A3 = anticipation phase; P1-P5 = performance phase.

**Figure S3: Difference of mean SUDS score and heart rate between male and female patients receiving placebo during the TSST.**

**A**





**B**

**
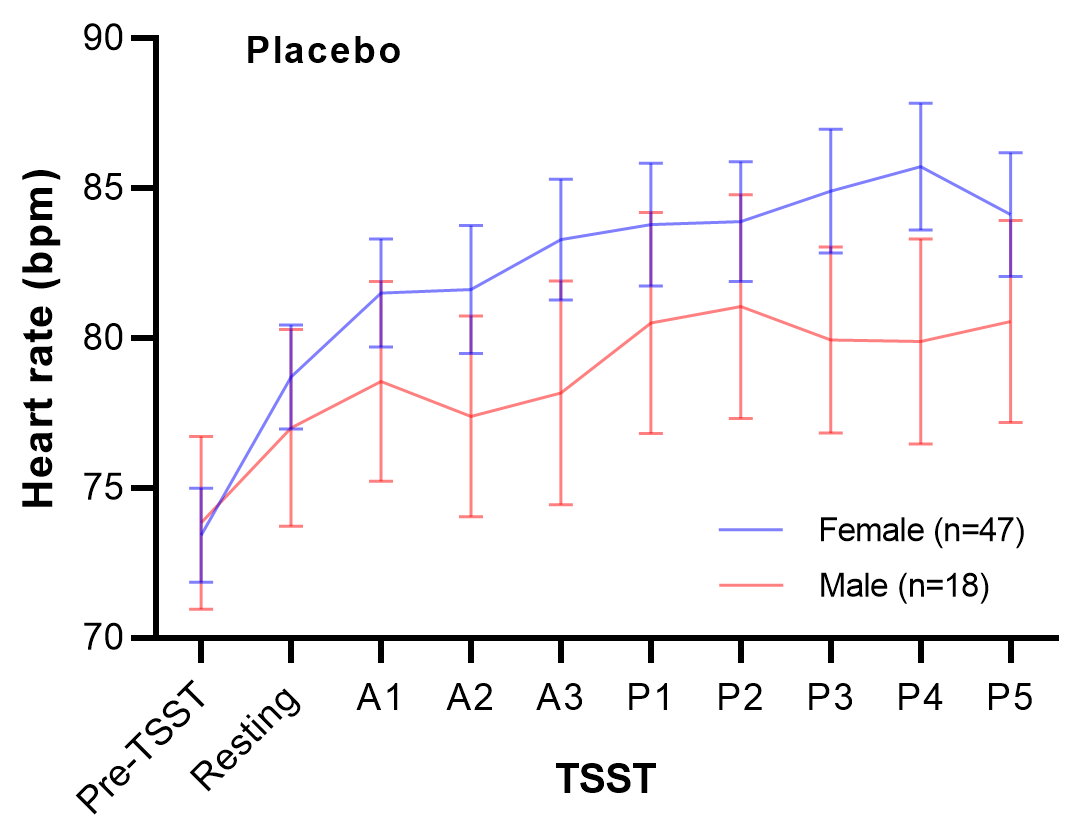
**

Curves represent mean SUDS score (A) and mean heart rate (B) in each timepoint of the TSST for male (red) and female (blue) patients treated with placebo. Baseline = pre-dose; Resting = resting phase (task introduction); A1-A3 = anticipation phase; P1-P5 = performance phase; * indicates significant difference between male and female with p-value <0.05.

**Figure S4: Plasma exposure of VQW-765 after a single oral dose of 10 mg VQW-765.**

VQW-765 concentration in plasma was measured after the TSST. The individual exposure is indicated by ♦. Based on the corresponding SUDS rating, the efficacious exposure range is between 1.5-8.0 pmol/mL (A). VQW-L, VQW-M, and VQW-H indicate low(blue), moderate (red), and high (yellow) exposure, respectively.

**Figure S5: Mean SUDS score for male patients receiving VQW-765 or placebo during the TSST.**


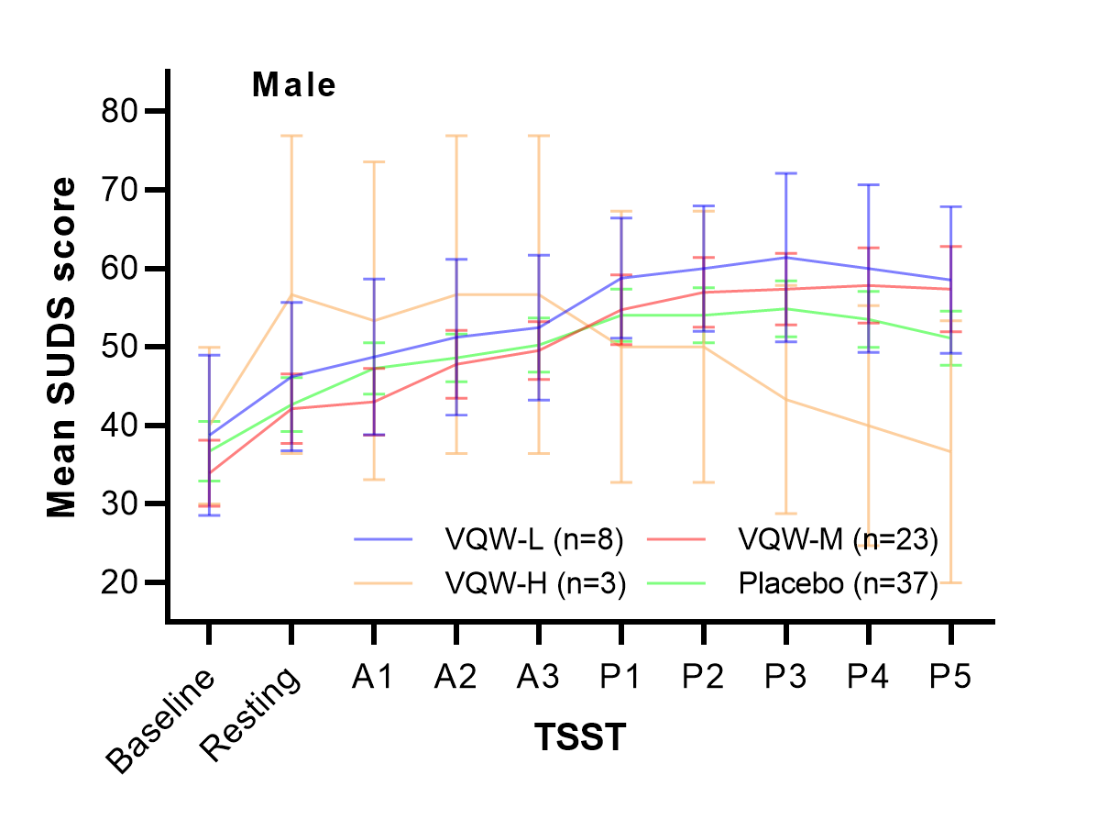


The curves represent mean SUDS score (± SEM) in each timepoint of the TSST for the male patients with different exposure of VQW-765 and placebo. VQW-L, VQW-M, and VQW-H indicate low, moderate, and high exposure, respectively. Baseline = pre-dose phase; Resting = resting phase (task introduction); A1-A3 = anticipation phase; P1-P5 = performance phase.

**Figure S6: Mean SUDS score during the TSST for patients with synchronized SUDS and HR data.**

**A**

**
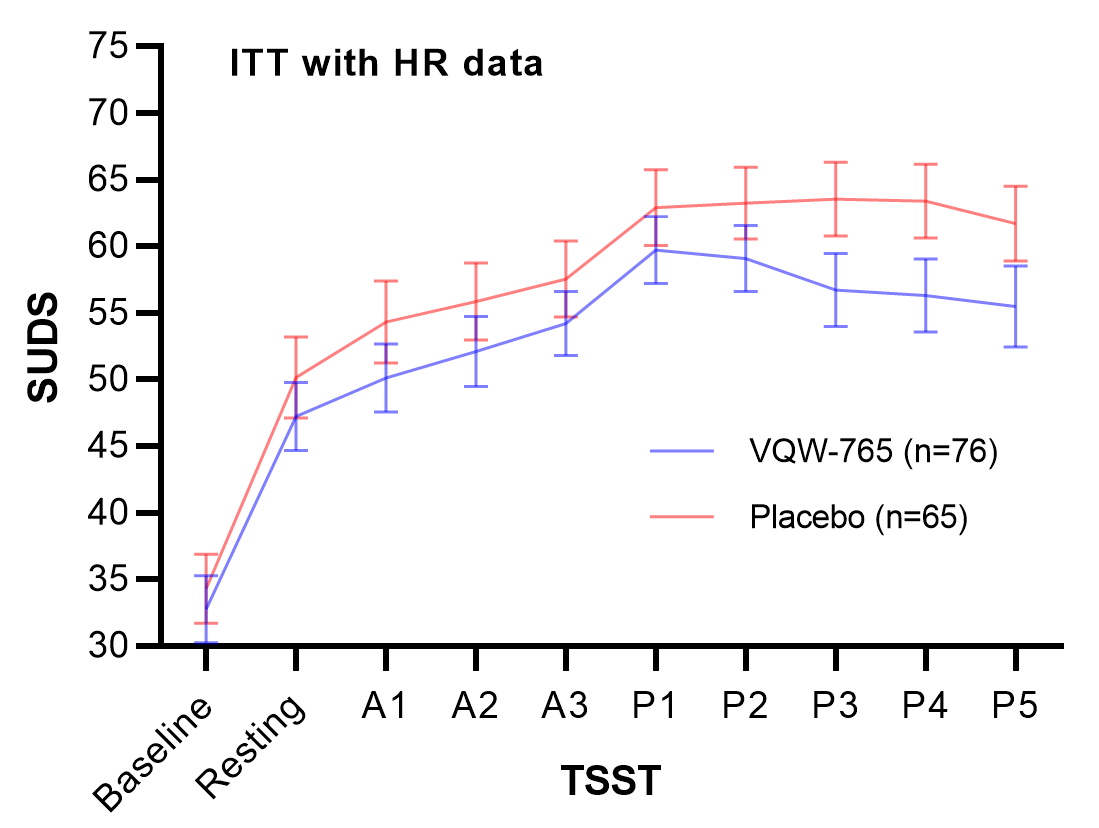
**

**B**


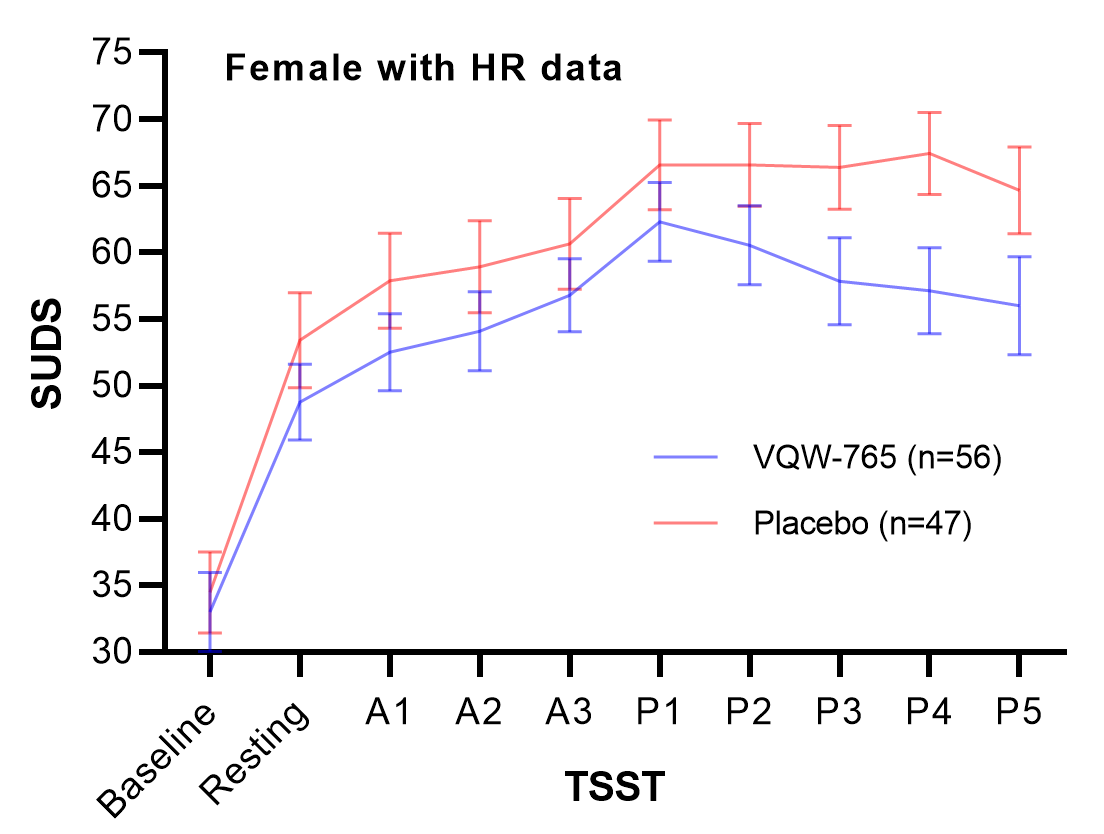


The mean SUDS scores (± SEM) in each timepoint of the TSST for groups treated with VQW-765 or placebo in the ITT (A) and Female (B) populations are illustrated. Baseline = pre-dose phase; Resting = resting phase (task introduction); A1-A3 = anticipation phase; P1-P5 = performance phase.

**Figure S7: Mean SUDS score of patients who did not wear face mask during the TSST.**





Curves represent mean SUDS score (± SEM) in each timepoint of the TSST for groups treated with VQW-765 (blue) or placebo (red). Baseline = pre-dose phase; Resting = resting phase (task introduction); A1-A3 = anticipation phase; P1-P5 = performance phase; * indicates significant difference between VQW-765 and placebo with p-value <0.05.

**Supplemental Tables.**

**Table S1: Exploratory efficacy analyses**


|  |  |  | **VQW-765 (n=69)** | | **Placebo (n=114)** | |  |
| --- | --- | --- | --- | --- | --- | --- | --- |
| **Population** | **Assessment** | **Phase** | **Mean** | **SE** | **Mean** | **SE** | **P-value** |
| **ITT - Efficacious exposure** | **SUDS** | **Pre-dose** | 35.51 | 2.83 | 37.02 | 2.19 | 0.3368 |
|  |  | **Resting** | 45.07 | 2.61 | 51.15 | 2.33 | 0.0471 |
|  |  | **Anticipation** | 49.86 | 2.5 | 56.9 | 2.06 | 0.0165 |
|  |  | **Performance** | 54.96 | 2.94 | 62.26 | 2.1 | 0.0202 |
|  | **CGI-C** | **Post-TSST** | 3.5 | 0.14 | 3.6 | 0.1 | 0.4343 |
|  | **PGI-C** | **Post-TSST** | 3.4 | 0.14 | 3.5 | 0.1 | 0.5525 |
|  |  |  |  |  |  |  |  |
|  |  |  | **VQW-765 (n=46)** | | **Placebo (n=77)** | | **P-value** |
| **Female - Efficacious exposure** | **SUDS** | **Pre-dose** | 36.3 | 3.72 | 37.14 | 2.7 | 0.4267 |
|  |  | **Resting** | 46.52 | 3.23 | 55.26 | 2.93 | 0.0282 |
|  |  | **Anticipation** | 51.38 | 3.2 | 60.88 | 2.56 | 0.0116 |
|  |  | **Performance** | 54 | 3.8 | 66.42 | 2.55 | 0.0029 |
|  | **CGI-C** | **Post-TSST** | 3.4 | 0.19 | 3.7 | 0.13 | 0.2417 |
|  | **PGI-C** | **Post-TSST** | 3.4 | 0.18 | 3.5 | 0.13 | 0.4655 |
|  |  |  |  |  |  |  |  |
|  |  |  | **VQW-765 (n=59)** | | **Placebo (n=92)** | | **P-value** |
| **ITT - Efficacious exposure (LSAS≥60)** | **SUDS** | **Pre-dose** | 37.46 | 2.95 | 39.78 | 2.5 | 0.3088 |
|  |  | **Resting** | 45.76 | 2.93 | 52.97 | 2.59 | 0.0293 |
|  |  | **Anticipation** | 50 | 2.9 | 57.62 | 2.33 | 0.0156 |
|  |  | **Performance** | 54.44 | 3.39 | 62.85 | 2.38 | 0.0149 |
|  |  |  |  |  |  |  |  |
|  |  |  | **VQW-765 (n=43)** | | **Placebo (n=64)** | | **P-value** |
| **Female - Efficacious exposure (LSAS≥60)** | **SUDS** | **Pre-dose** | 37.44 | 3.74 | 40.31 | 3.04 | 0.2844 |
|  |  | **Resting** | 46.74 | 3.5 | 56.83 | 3.19 | 0.019 |
|  |  | **Anticipation** | 51.09 | 3.6 | 61.69 | 2.81 | 0.0103 |
|  |  | **Performance** | 54.23 | 4.14 | 66.84 | 2.8 | 0.0048 |

Efficacious exposure = 1.5-8.0 pmol/mL of VQW-765 plasma concentration; SUDS = Subjective Units of Distress Scale; TSST = Trier Social Stress Test; CGI-C = Clinician Global Impression of Change; PGI-C = Patient Global Impression of Change.

**Table S2: Summary for treatment emergent adverse events**

| **Treatment Emergent Adverse Events (TEAE)** | **VQW-765 (n=116)** | **Placebo (n=114)** | **Overall (n=230)** |
| --- | --- | --- | --- |
|  | **n (%)** | **n (%)** | **n (%)** |
|  | | | |
| **Subjects with any TEAE** | 6 (5.2) | 13 (11.4) | 19 (8.3) |
|  | | | |
| **Headache** | 2 (1.7) | 4 (3.5) | 6 (2.6) |
| **Alanine aminotransferase increased** | 2 (1.7) | 1 (0.9) | 3 (1.3) |
| **Blood thyroid stimulating hormone increased** | 0 | 2 (1.8) | 2 (0.9) |
| **Nausea** | 0 | 2 (1.8) | 2 (0.9) |
| **Abdominal discomfort** | 1 (0.9) | 0 | 1 (0.4) |
| **Dry mouth** | 0 | 1 (0.9) | 1 (0.4) |
| **Chest discomfort** | 1 (0.9) | 0 | 1 (0.4) |
| **Fatigue** | 1 (0.9) | 0 | 1 (0.4) |
| **Feeling abnormal** | 0 | 1 (0.9) | 1 (0.4) |
| **Bundle branch block right** | 0 | 1 (0.9) | 1 (0.4) |
| **Tachypnoea** | 1 (0.9) | 0 | 1 (0.4) |
